# Editorial: Bias, Subjectivity and Perspectives in Natural Language Processing

**DOI:** 10.3389/frai.2022.926435

**Published:** 2022-05-24

**Authors:** Valerio Basile, Tommaso Caselli, Alexandra Balahur, Lun-Wei Ku

**Affiliations:** ^1^Computer Science Department, University of Turin, Turin, Italy; ^2^CLCG, University of Groningen, Groningen, Netherlands; ^3^European Commission Joint Research Centre, Brussels, Belgium; ^4^Institute of Information Science, Academia Sinica, Taipei, Taiwan

**Keywords:** perspectives, subjectivity, Data-centric AI, bias, NLP

Subjectivity represents a core and pervasive element of human life and way of thinking. Reality, in spite of having been thought for a long time in terms as transcendent and objective, with all humans being able to access the same version of it, has in fact been shown by Cognitive Science to be a collection of perspectives. Moreover, thoughts as cognitive processes, are embodied (are linked to the body experiencing it and its conditions), imaginative (depend on prior experience and imagination potential), have gestalt (the brain tends to complete missing information with most plausible/available information) and has ecological structure (it depends on the environment) (Lakoff, 1987).

As such, while we can communicate and share information with a wide range of people, the way people write and speak about events, entities, and their experiences is unique and subjective. The same is true for the recipients of communication that interpret the meaning of messages based on their own perspective - i.e. understanding, experience and states. The easiest way in which this pervasiveness of subjectivity can be observed is at the lexical level: the choice of words definitely reflects social and personal perspectives, opinions, and stances (Lin et al., 2006). Other work has shown that more subtle ways of expressing perspectives in discourse can be found at the syntax-semantic interface (Horst, 2020; Te Brömmelstroet, 2020; Pinelli and Zanchi, 2021; Minnema et al., 2022). While most work in Computational Linguistics and Natural Language Processing still focuses on language phenomena on which humans agree to a large extent, there is a growing interest in modeling people's perspectivization of a shared event or language phenomenon by taking into account their own interests, agenda, and how that influences the way in which communication is perceived. To achieve this goal, a new generation of language resources is needed. Additionally, recent advancements thanks to large pre-trained language models (PTLMs), dedicated resources are necessary to apply these models to perspective-oriented tasks via fine-tuning.

The creation of language resources is always a challenging task. Although accompanied by annotation guidelines, annotated data will always contain some forms of bias. Awareness of bias in the data (and consequently, in the trained models) is growing in the NLP community with dedicated efforts to capture, remove, and understand the effect of bias in the data and on the prediction capabilities of the models (Bolukbasi et al., 2016; Bender and Friedman, 2018; Gonen and Goldberg, 2019; Bartl et al., 2020; Bender et al., 2021). Efforts are made in various fora to understand and mitigate causes of bias in the data, especially in the data employed in AI models, as they represent the future of software applications in many fields. At the same time, bias and disagreements represent a key source of information when modeling subjectivity and perspectives. One of the potential end goals here is to be able to make explicit the different perspectives of speakers with respect to a common target (being it an entity or an event) so as to gain a more comprehensive understanding of their opinions, attitudes, and the impacts on their life. If this is the ultimate goal, disagreements in annotations are more informative than one can imagine. Previous work has already shown how disagreements can be used in an effective way to extract more fine-grained information (Aroyo and Welty, 2015). While mining the disagreement for additional knowledge is a difficult task (Basile et al., 2021), the benefits may surpass the challenges (Davani et al., 2021). Finally, being able to detect bias in data or in AI models can support understanding bias in society and where further efforts must be made to raise awareness about it, as well as work on strategies to mitigate it. AI can be seen as a mirror of society, both to inform on better strategies to accomplish tasks in a personalized manner (e.g. in recommender systems, personalized medicine) or detect potential discrimination and socially unfair situations.

The adoption of a perspectivist approach both in the creation of language resources and the development of new models is the necessary step forward to address these challenges and present a modelization of subjectivity and users' perspectives which is able to capture multiple points of view encoding different cultural and personal backgrounds. Recent initiatives such as the workshop on “Benchmarking: Past, Present and Future” at ACL 2021^1^, and the 1st workshop on “Perspectivist Approaches to Natural Language Processing” at LREC 2022^2^ are all initial steps showing a growing interest in this approach within the NLP and AI communities.

This Research Topic presents an initial collection of contributions that investigates bias, perspectives, and subjectivity across different topics and using different approaches.

Rao and Taboada apply a mixture of techniques from NLP, data analysis, and visualization, to study the bias in English newswire text. Leveraging 2 years of data, the findings of the paper indicate how gender bias in the news is powered by a self-reinforcing loop, where consistent mentions of people in traditional gender roles leads to the consolidation of the bias itself. Therefore, solutions are needed to support the monitoring and correction of this phenomenon.

Bias is in the eye of the speaker (or writer), but also in that of the hearer (or reader). In their contribution to, Dönicke et al. show how different annotators of literary texts tend to attribute different speakers to passages based on their own background and beliefs.

Training datasets containing biases are bound to produce biased models of language. This can have undesirable consequences when the bias model becomes part of user-facing applications, such as Automated Speech Recognition, as shown by the article by Mengesha et al. Specifically, users from minority groups, such as speakers of African American Vernacular English, are consistently and negatively impacted by errors in ASR more than other socio-demographic groups, with detrimental consequences for their psychological wellbeing.

In conclusion, in the current era of NLP data are becoming more and more pivotal both to model new tasks and to develop perspective-aware models. The challenges are many, whether involving new strategies to mine relevant linguistic phenomena from large corpora such as expressions of stereotypes (see the contribution to this Research Topic by Fraser et al., or balancing the over-representation of some languages in the research community with respect to less-resourced languages such as Arabic (see the article by Alqahtani and Alothaim).

The issues raised in this Research Topic, together with initiatives such as Data Statements^3^, Data-centric AI^4^, and the Perspectivist Data Manifesto^5^, all confirm the importance of the focus on the quality, fairness, and availability of data for the next generation of NLP systems.

## Author Contributions

All authors listed have made a substantial, direct, and intellectual contribution to the work and approved it for publication.

## Conflict of Interest

The authors declare that the research was conducted in the absence of any commercial or financial relationships that could be construed as a potential conflict of interest.

## Publisher's Note

All claims expressed in this article are solely those of the authors and do not necessarily represent those of their affiliated organizations, or those of the publisher, the editors and the reviewers. Any product that may be evaluated in this article, or claim that may be made by its manufacturer, is not guaranteed or endorsed by the publisher.

## Footnotes

^1^ https://github.com/kwchurch/Benchmarking_past_present_future

^2^ https://nlperspectives.di.unito.it/

^3^ https://techpolicylab.uw.edu/events/event/data-statements-for-nlp/

^4^ https://sites.google.com/view/dataperf2022

^5^ https://pdai.info/

## References

[B1] Aroyo L. Welty C. (2015). Truth is a lie: Crowd truth and the seven myths of human annotation. AI Magaz. 36, 15–24. 10.1609/aimag.v36i1.2564

[B2] Bartl M. Nissim M. Gatt A. (2020). Unmasking contextual stereotypes: Measuring and mitigating BERT's gender bias. arXiv preprint arXiv:2010.14534.

[B3] Basile V. Fell M. Fornaciari T. Hovy D. Paun S. Plank B.. (2021). “We need to consider disagreement in evaluation”, in 1st Workshop on Benchmarking: Past, Present and Future. Association for Computational Linguistics. p. 15–21. 10.18653/v1/2021.bppf-1.3

[B4] Bender E. M. Friedman B. (2018). Data statements for natural language processing: toward mitigating system bias and enabling better science. Transac. Assoc. Comput. Ling. 6, 587–604. 10.1162/tacl_a_00041

[B5] Bender E. M. Gebru T. McMillan-Major A. Shmitchell S. (2021). “On the dangers of stochastic parrots: can language models be too big?”, in Proceedings of the 2021 ACM Conference on Fairness, Accountability, and Transparency. p. 610–623. 10.1145/3442188.3445922

[B6] Bolukbasi T. Chang K. W. Zou J. Y. Saligrama V. Kalai A. T. (2016). “Man is to computer programmer as woman is to homemaker? Debiasing word embeddings”, in 30th Conference on Neural Information Processing Systems (NIPS 2016). p. 1–9. Barcelona, Spain.

[B7] Davani A. M. Díaz M. Prabhakaran V. (2021). Dealing with disagreements: looking beyond the majority vote in subjective annotations. arXiv preprint arXiv:2110.05719. 10.1162/tacl_a_00449

[B8] Gonen H. Goldberg Y. (2019). Lipstick on a pig: debiasing methods cover up systematic gender biases in word embeddings but do not remove them. arXiv preprint arXiv:1903.03862. 10.18653/v1/N19-1061

[B9] Horst D. (2020). Patterns ‘we' think by? Critical cognitive linguistics between language system and language use. Yearb. German Cogn. Ling. Assoc. 8, 67–82. 10.1515/gcla-2020-0005

[B10] Lakoff G. (1987). “Cognitive models and prototype theory,” in Concepts and Conceptual Development: Ecological and Intellectual Factors in Categorization, ed U. Neisser (Cambridge University Press), 63–100.

[B11] Lin W. H. Wilson T. Wiebe J. Hauptmann A. G. (2006). “Which side are you on? Identifying perspectives at the document and sentence levels”, in Proceedings of the Tenth Conference on Computational Natural Language Learning (CoNLL-X), New York NY: Association for Computational Linguistics. p. 109–116. 10.3115/1596276.1596297

[B12] Minnema G. Gemelli S. Zanchi C. Caselli T. Nissim M. (2022). SOCIOFILLMORE: a tool for discovering perspectives. arXiv preprint arXiv:2203.03438.

[B13] Pinelli E. Zanchi C. (2021). “Gender-based violence in italian local newspapers: how argument structure constructions can diminish a perpetrator's responsibility”, in Discourse Processes between Reason and Emotion. Palgrave Macmillan: Cham. p. 117–143. 10.1007/978-3-030-70091-1_6

[B14] Te Brömmelstroet M. (2020). Framing systemic traffic violence: media coverage of Dutch traffic crashes. *Transp. Res. Interdiscipl*. Perspect. 5, 100109. 10.1016/j.trip.2020.100109

